# False negative results from using common PCR reagents

**DOI:** 10.1186/1756-0500-4-457

**Published:** 2011-10-27

**Authors:** Dean J Bacich, Kathryn M Sobek, Jessica L Cummings, Allison A Atwood, Denise S O'Keefe

**Affiliations:** 1 Department of Urology, University of Pittsburgh, 5200 Centre Avenue, Pittsburgh, PA 15232, USA

## Abstract

**Background:**

The sensitivity of the PCR reaction makes it ideal for use when identifying potentially novel viral infections in human disease. Unfortunately, this same sensitivity also leaves this popular technique open to potential contamination with previously amplified PCR products, or "carry-over" contamination. PCR product carry-over contamination can be prevented with uracil-DNA-glycosylase (UNG), and it is for this reason that it is commonly included in many commercial PCR master-mixes. While testing the sensitivity of PCR assays to detect murine DNA contamination in human tissue samples, we inadvertently discovered that the use of this common PCR reagent may lead to the production of false-negative PCR results.

**Findings:**

We show here that contamination with minute quantities of UNG-digested PCR product or any negative control PCR reactions containing primer-dimers regardless of UNG presence can completely block amplification from as much as 60 ng of legitimate target DNA.

**Conclusions:**

These findings could potentially explain discrepant results from laboratories attempting to amplify MLV-related viruses including XMRV from human samples, as none of the published reports used internal-tube controls for amplification. The potential for false negative results needs to be considered and carefully controlled in PCR experiments, especially when the target copy number may be low - just as the potential for false positive results already is.

## Findings

Carry-over contamination can be prevented by incorporating uracil instead of thymine into the PCR product, then treating with uracil-DNA-glycosylase (UNG) prior to initiating subsequent PCR reactions [1]. The UNG degrades DNA containing uracil, thereby degrading contaminating PCR products while leaving non-uracil DNA (ie. target DNA) intact. This method is so effective that several major brands of Taq and Taq master-mixes include this enzyme and replace dTTP with dUTP. In addition the enzyme is sold separately and as part of kits designed to prevent carry-over contamination.

Xenotropic Murine leukemia virus Related Virus (XMRV) was isolated from patients with prostate cancer in 2006, and since that time many reports have either identified XMRV or the highly related murine leukemia viruses (MLVs) in various patient cohorts. However, many reports have been unable to find either of these types of viruses. Mice, particularly strains of mice used in laboratory research, carry 20-40 copies of MLV-type viruses. Therefore MLV-type viral DNA detected in patient samples could be due to actual MLV infection of patient tissue or contamination of patient samples with mouse DNA. We developed an assay to detect femtogram level contamination of murine DNA in human DNA samples. This sensitivity is necessary because MLV-related viruses found in human populations seem to be present at very low levels. Therefore low levels of murine DNA contamination could give rise to false-positive results.

### Use of Uracil-DNA-glycosylase inhibits amplification of legitimate target DNA in the presence of low levels of PCR carry-over contamination

**Figure 1:**
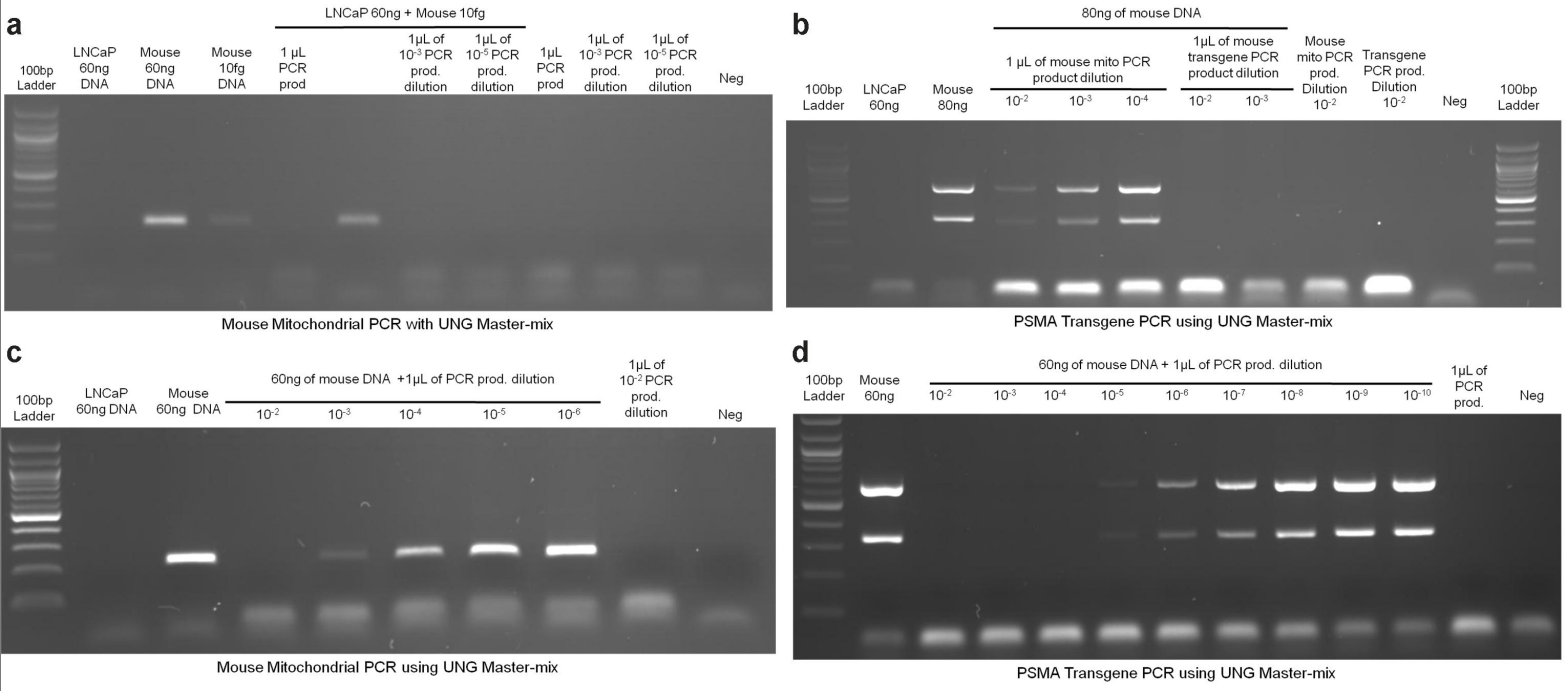
**PCR contamination inhibits legitimate target amplification**. a) PCR to amplify mouse mitochondrial DNA was optimized to detect from 60 ng to 10 fg of mouse DNA. The reaction was intentionally contaminated with previously generated mouse mitochondrial DNA PCR product. b) The PSMA mouse transgene PCR was intentionally contaminated with PCR products from the mouse mitochondrial DNA PCR and the PSMA PCR. c) Mouse mitochondrial PCR product was diluted from 10^-2 ^to 10^-6^. These dilutions were used to contaminate a subsequent mouse mitochondrial PCR. d) The PSMA PCR product was diluted from 10^-2 ^to 10^-10^. The dilutions were used to contaminate a subsequent PSMA PCR. Human LNCaP DNA and water were run as negative controls and 60 ng of mouse DNA was used as a positive control. All PCR products, including the PCR products used to contaminate the reactions, were amplified with ABI Gene Expression Master-mix containing UNG. The results demonstrate inhibition of amplification of large copy numbers of legitimate PCR target when contamination with minute quantities of the same or different previously generated PCR product was present.

We developed an assay to detect contaminating mouse DNA in human tissue samples using the Gene Expression master-mix from Applied Biosystems (Life Technologies Corp.). This master-mix is routinely employed by labs that carry out Taqman gene expression assays, and it contains uracil-DNA-glycosylase and dUTP to prevent PCR carry-over contamination. Elimination of any potential PCR product contamination is achieved by a two-minute incubation at 50°C prior to inactivation of the enzyme during the initial denaturation of the PCR reaction. When testing to confirm that PCR carry-over would be eliminated, we inadvertently discovered that contamination with as little as 10 picoliters of PCR product was able to inhibit amplification of legitimate, albeit low copy number target (Figure 1a). To determine both the specificity as well as the effectiveness of the inhibition of amplification of legitimate target, we intentionally contaminated mouse DNA with PCR product generated using the Gene Expression master-mix. As shown in Figure 1b, when we attempted to amplify two unrelated targets from 80 nanograms of mouse DNA, deliberate contamination with a third unrelated PCR product (mouse mitochondrial DNA), resulted in significant inhibition of amplification, even when the amount of PCR product added was just one nanoliter. Furthermore, when PCR product that was the same as the target reaction was deliberately spiked into the DNA samples and the PCR was performed with a master-mix containing UNG, it almost *completely *inhibited amplification from 60 ng of legitimate target, even at a final dilution of 1:25, 000 and 1:25 million (Figure 1c and 1d, respectively). Therefore accidental cross-contamination with previously generated PCR product can significantly inhibit legitimate target amplification, even when the true target is present at a very high copy number, and the contamination is minute.

### Primer-dimers from previously generated reactions can also inhibit legitimate target amplification, regardless of the presence of uracil-DNA-glycosylase

**Figure 2:**
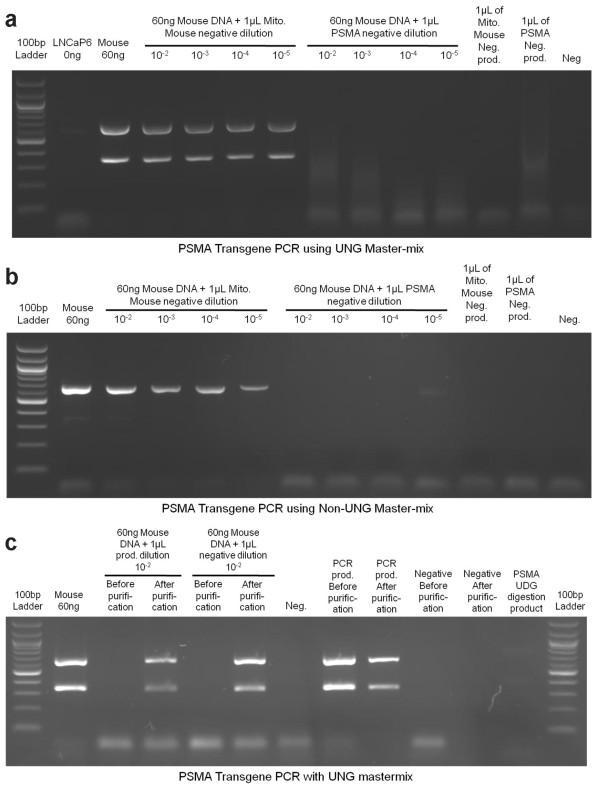
**Primer-dimers and UNG degradation products can inhibit PCR amplification**. a) The negative control reactions (water and PCR reagents, but no DNA) from the mouse mitochondrial DNA PCR and the PSMA transgene PCR were diluted from 10^-2 ^to 10^-5^. Both sets of dilutions were used to contaminate a PCR for the PSMA transgene. All reactions were amplified with the ABI Gene Expression Master-mix (containing UNG). b) The same experiment was conducted except the NEB Taq Polymerase and buffer was used (no UNG in the PCR for both generation of the primer-dimers and subsequent PCR). NEB Taq is less efficient than ABI Taq at amplifying the target for the lower band in the PSMA transgene PCR, however inhibition by contaminating primer-dimers is the same regardless of PCR mix. c) The PSMA transgene PCR product and negative control were amplified with the ABI Gene Expression Master-mix and gel purified to remove primer-dimers. These products as well as non-purified PSMA transgene PCR product and negative control (that contain primer-dimers) were used to contaminate a subsequent PSMA transgene PCR. The results indicate that amplification of legitimate PCR target is significantly inhibited by contamination with previously generated negative control reactions using the same primers, regardless of the presence or absence of UNG. It also indicates that the UNG degradation products can weakly inhibit amplification and the UNG degradation products and primer-dimers can completely inhibit PCR amplification.

Presumably inhibition of the PCR reaction is due to the competition for genomic DNA target binding between the primers and degraded PCR products. Based upon this reasoning we tested if primer-dimers generated from a negative control PCR reaction could inhibit subsequent PCR amplification. We performed this experiment using a master-mix with and without UNG, hypothesizing that the presence of primer-dimers may be sufficient to inhibit PCR amplification. For this experiment we intentionally contaminated mouse DNA with PCR primer-dimers generated from a PCR reaction using either a UNG- or non-UNG-containing master-mix. The negative PCR reaction was able to inhibit the subsequent PCR reaction at a 10^-5 ^dilution if the same primers were used in the subsequent reaction, but they did not inhibit amplification of an unrelated target (Figure 2a). There was no difference between UNG and non UNG master-mixes (Figure 2a and 2b). Given that the primer-dimers of the negative PCR reaction were able to effectively inhibit subsequent PCR reactions, we then tested if it was just the primer-dimers in the UNG containing PCR that provide the inhibition or if the degraded PCR amplicon was also capable of inhibiting the subsequent PCR reaction. By gel isolating the UNG incorporated PCR product to remove the primer-dimers, and then spiking (10^-2 ^dilution) this into a fresh PCR we were able to demonstrate that this weakly inhibited the subsequent PCR (Figure 2c).

### Primer dimers from previously generated reactions demonstrate a strong inhibitory effect on legitimate target amplification of an MLV-carrying plasmid

**Figure 3:**
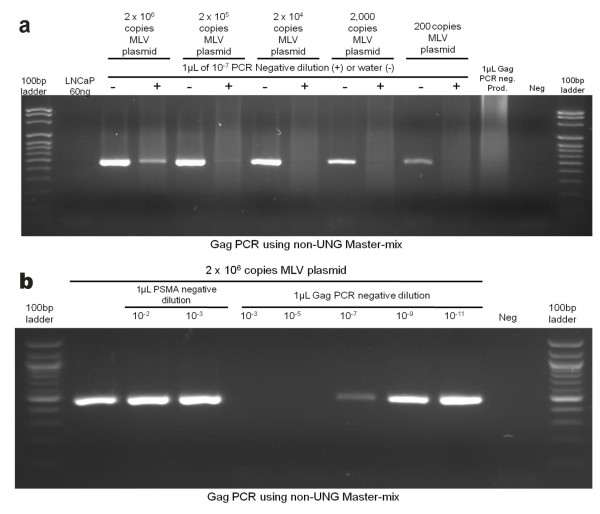
**Primer-dimers from previously generated reactions demonstrate a strong inhibitory effect on legitimate target amplification of MLV Gag region**. a) The negative control reactions (water and PCR reagents but no DNA) from the PSMA PCR and MLV Gag PCR were diluted as shown. Both sets of dilutions were used to contaminate a PCR for MLV Gag. The MLV Gag PCR used Titanium Taq Polymerase and buffer and the PSMA PCR used NEB Taq Polymerase and buffer, with both PCRs being non-UNG containing. b) A negative control reaction (water and PCR reagents but no DNA) from the MLV Gag PCR was diluted 1 × 10^-7^. This PCR, which used non-UNG containing Titanium Taq Polymerase and buffer, was used to contaminate a subsequent MLV Gag PCR. The MLV-plasmid was used in decreasing copy number, as indicated.

Next we wanted to examine the extent of primer-dimer inhibition of PCR product formation when specifically amplifying MLVs. We performed a PCR on plasmid DNA containing the Gag region of an MLV using primers within this Gag region. We started with 2 × 10^6 ^copies (6 pg) of plasmid DNA and contaminated this PCR with different dilutions of either unrelated target primer-dimers (PSMA transgene) or Gag PCR primer-dimers from previous PCR reactions. As seen in Figure 3a, the unrelated target primer-dimers do not inhibit PCR product formation whereas contamination with Gag PCR primer-dimers completely inhibits PCR product formation up to 10^-5 ^dilution and demonstrates weak inhibition of PCR product formation through 10^-9 ^dilution.

MLVs previously found to be present in patient samples have been observed at very low levels. Therefore we wanted to examine the inhibitory effect that minute levels of primer-dimer contamination (10^-7 ^dilution) can have on Gag PCR product formation from MLVs that are present at low copy number. Figure 3b demonstrates that contamination of MLV present at 200, 000 copies or less with 10^-7 ^dilution of Gag PCR primer-dimers from a previous PCR reaction almost completely inhibits PCR product formation. This data suggests that contamination with extremely small quantities of primer-dimers generated from previous PCR reactions can inhibit PCR product formation, thus leading to false negative results.

Taken together, these data suggest that inhibition of legitimate target occurs from UNG-digested PCR products or from contamination with primer-dimers from a previous PCR reaction, regardless of the presence of UNG. Thus contamination from any previous PCR samples can result in either false positives (which may or may not be detected in the negative control depending upon the samples that are contaminated and whether UNG is used) or false negatives which will not be detected using standard PCR controls.

## Discussion and Conclusions

As these results could potentially explain some of the discrepancies in amplification of MLV-related viruses from different laboratories, we surveyed these publications to attempt to determine if the authors used UNG-containing master-mixes. Of concern, despite the importance of the actual PCR methods used to amplify these potentially novel viruses, we were unable to determine the type of Taq or master-mix used in 11 of 38 publications. Of the remaining 27 publications, 13 studies used Taq or master-mixes likely containing UNG (it was present in 8 studies [2-9] and possibly used in the remaining 5 studies [10-14] that we examined). Therefore contamination at either the individual sample level or in the PCR reaction itself could have inhibited amplification of legitimate target, especially if the target is found at low levels. Additionally, as shown in Figure 2, regardless of the presence of UNG, PCR can be inhibited if there is even a minute amount of contamination from previous (negative) PCR reactions.

Based on our data, we could speculate that low levels of PCR product contamination may not be sufficient to significantly block amplification of the positive control in these reactions, especially if the positive control is the target cloned into a plasmid and present at a high copy number. In the majority of the studies commented on in this manuscript, the positive control used was an XMRV plasmid, and most papers were not clear about the amount of plasmid used for control amplification. Therefore, amplification of the high copy number positive control could occur while samples positive for a low copy number virus could be inhibited by carry-over PCR contamination.

To circumvent the potential for PCR inhibition, a positive control included in each PCR reaction is necessary to confirm that the PCR is not inhibited. To accomplish this, a synthetic target template with identical primer-binding sites but different size could be constructed, and spiked into each test sample. This would result in potential amplification of two bands: one band to detect the target DNA and the other to detect the synthetic target. In addition, the copy number of the synthetic target spiked into the test samples would need to be at the level of detection of the assay, so as any inhibition would result in lack of amplification. None of the 38 MLV-related publications we examined used internal PCR controls. Given the data presented in this manuscript, we would propose that in cases such as the debate regarding the existence of novel infectious viruses, failure to find a virus is not the equivalent of evidence against its existence. The potential for false negative results needs to be considered and carefully controlled in PCR experiments, just as the potential for false positive results already is.

## Methods

### DNA extraction

Human LNCaP cell line DNA was extracted with the DNeasy^® ^Blood & Tissue Kit (Qiagen). DNA from mouse tails was extracted with the Wizard SV Genomic DNA purification system (Promega). Approval for mouse work was given by the University of Pittsburgh IACUC, under protocol #1991385A, and the work was performed according to internationally accepted guidelines. In order to remove primer-dimers from the PSMA PCR product and negative control, the bands of the PCR product and negative control were cut from a 0.8% agarose gel. The GENECLEAN^® ^II Kit (Qbiogene) was used to purify the samples per manufacturer's instructions. Five microliters of the isolation products were electrophoresed on a 1.5% agarose gel to confirm the purified PCR and lack of primer-dimers.

### Mouse mitochondrial DNA, PSMA transgene, and MLV-Gag PCR

All primer sequences are listed in Table 1.

**Table 1:** Primer sequences used in these experiments.

Primer Name	Primer sequence
Mouse mito F	5'-ACTATCCCCTTCCCCATTTG-3'

Mouse mito R	5'-TGTTGGTCATGGGCTGATTA-3'

S49	5'-CAGATATGTCATTCTGGGAGG-3'

Inta	5'-GTAGAAGAGAACTGCTGAGGA-3'

S1368	5'-ATTCAATCCTGCTCAGACCC-3'

AS2015	5'-AACACCATCCCTCCTCGAACC-3'

MLV Gag F	5'-CCTTGGGAGGGTCTCCTCAG-3'

MLV Gag R	5'-CAGA CGCGCCGCGCGGTTTC-3'

Mitochondrial PCR: 60 ng of mouse DNA was amplified in a 25 μL reaction containing 12.5 μL 2× Gene Expression Master-Mix (Applied Biosystems), 0.5 μM Mouse mito F primer, 0.5 μM Mouse mito R primer, and 9.5 μL of water. Two microliters of 30 ng/μL mouse DNA was added the reaction. The following cycle conditions were used: 50°C for 2 minutes (UNG incubation), 94°C for 10 minutes (AmpliTaq Gold Enzyme Activation), and 40 cycles of 95°C for 15 seconds and 60°C for 1 minute. The reactions were carried out in an Eppendorf Mastercycler personal thermocycler. The expected size of the PCR product is ~230 bp. PCR reactions were run on a 1.5% agarose/TBE gel.

Gag PCR: Varying amounts of plasmid DNA was amplified in a 25 μL reaction containing 2.5 μL 10× Titanium Taq PCR Buffer (Clonetech), 0.2 uL Titanium Taq (Clonetech), 0.2 mM dNTPs, 0.5 μM MLV Gag F primer, 0.5 μM MLV Gag R primer, and 19.8 μL of water. The following cycle conditions were used: 94°C for 1 minutes, 40 cycles of [95°C for 30 seconds and 68°C for 30 seconds], 68°C for 3 minutes. The reactions were carried out in an Eppendorf Mastercycler personal thermocycler. The expected size of the PCR product is ~500 bp. PCR reactions were run on a 1.4% agarose/TBE gel.

PSMA transgene PCR: The PSMA PCR contains 2 sets of primers to amplify two unrelated targets from PSMA transgenic mice (the transgene and genomic control sequence). Sixty to eighty nanograms of transgenic mouse DNA was amplified in a 25 μL reaction containing 12.5 μL 2× Gene Expression Master-Mix (Applied Biosystems), 1 μM S49, 1 μM INTA, 1 μM S1368, 1 μM AS2015, 6.5 μL of water, and 2 μL 30 ng/μl to 40 ng/μL mouse DNA. The following cycle conditions were used: 50°C for 2 minutes (UNG incubation), 94°C for 10 minutes (AmpliTaq Gold Enzyme Activation), 35 cycles of 94°C for 30 seconds, 58°C for 30 seconds, and 72°C for 45 seconds. The expected product sizes are ~600 bp and ~250 bp and were electrophoresed on a 1.5% agarose/TBE gel.

The PSMA PCR was also amplified with non-UNG containing taq. Sixty nanograms of transgenic mouse DNA was amplified in 25 μL reaction containing 0.5 U Taq DNA Polymerase, 10× ThermoPol Reaction Buffer (New England BioLabs, Inc.), 0.4 mM dNTP (dATP, dTTP, dCTP, and dGTP), 1 μM S49, 1 μM INTA, 1 μM S1368, and 1 μM AS2015. The following cycle conditions were used: 94°C for 5 minutes, 35 cycles of 94°C for 30 seconds, 58°C for 30 seconds, and 72°C for 45 seconds.

Negative PCR products (only water added to the PCR reaction) of the mouse mitochondrial PCR, PSMA PCR, and Gag PCR were also diluted 10^-2 ^through 10^-11 ^and used to simulate contamination of subsequent PCR reactions. PCR products ranged from 4 ng/μl (PSMA PCR lower band) to 10 ng/μl for all other reactions, prior to dilution for use in simulated contamination experiments.

## Competing interests

The authors declare that they have no competing interests.

## Authors' contributions

DJB conceived of the study, participated in its design and interpretation of results, and helped to draft the manuscript. KMS, JLC, and AAA carried out the molecular studies, participated in the design of the study and helped draft the manuscript. DOK participated in the design of the study, co-ordination of the experiments, analysis and interpretation and drafting the manuscript. All authors have read and approved the final version of this manuscript.
